# Immune suppressive activities of low-density neutrophils in sepsis and potential use as a novel biomarker of sepsis-induced immune suppression

**DOI:** 10.1038/s41598-025-92417-7

**Published:** 2025-03-19

**Authors:** Awirut Charoensappakit, Kritsanawan Sae‑khow, Nuntanuj Vutthikraivit, Patinya Maneesow, Thitiwat Sriprasart, Monvasi Pachinburavan, Asada Leelahavanichkul

**Affiliations:** 1 Medical Microbiology, Interdisciplinary and International Program, Graduate School, Chulalongkorn University, Bangkok, Thailand; 2 Center of Excellence On Translational Research in Inflammation and Immunology (CETRII), Department of Microbiology, Faculty of Medicine, Chulalongkorn University, 1873 King Rama 4 Road, Pathumwan, Bangkok, 10330 Thailand; 3 Division of Critical Care Medicine, Department of Medicine, Faculty of Medicine, Chulalongkorn University and King Chulalongkorn Memorial Hospital, The Thai Red Cross Society, Bangkok, Thailand; 4 Division of Respiratory Diseases and Critical Care Medicine, Department of Medicine, Faculty of Medicine, Chulalongkorn University and King Chulalongkorn Memorial Hospital, The Thai Red Cross Society, Bangkok, Thailand; 5 Division of Nephrology, Department of Medicine, Faculty of Medicine, Chulalongkorn University, Bangkok, Thailand; 6 Immunology Unit, Department of Microbiology, Faculty of Medicine, Chulalongkorn University, Bangkok, Thailand; 7 Department of Clinical Microscopy, Faculty of Allied Health Sciences, Chulalongkorn University, Bangkok, Thailand

**Keywords:** Infectious diseases, Innate immunity

## Abstract

Data of low-density neutrophils (LDN), the neutrophils in the peripheral blood mononuclear cells (PBMC) fraction, in sepsis is still less. As such, LDN (CD66b-positive cells in PBMC) was highest in intensive care unit (ICU) patients with sepsis (n=24) compared with non-sepsis (n=10) and healthy control (n=20), with a negative correlation with lymphocyte count and could predict secondary infection and mortality with the area under the curve (AUC) at 0.79 and 0.84, respectively. Compared with sepsis normal-density neutrophils (NDN), sepsis-LDN demonstrated higher expression of CD66b, CD63, CD11b, and CD184, but lower expression of CD62L and CD182 and defects of effector functions, including phagocytosis and apoptosis. The t-distributed stochastic neighbor embedding (t-SNEs) demonstrated high program cell death ligand-1 (PD-L1) in sepsis-LDN. In sepsis samples, the T cell proliferation in PBMC (T cells with LDNs) was lower than that in the isolated T cells (T cells alone) and incubation of anti-PD-L1 neutralizing antibody, but not a reactive oxygen species (ROS) scavenger (N-acetyl cysteine), improved the T cell suppression. Additionally, 30 min lipopolysaccharide (LPS) activation altered healthy control NDN into LPS-LDN (reduced density) and LPS-NDN (maintain density) with similarly elevated CD66b, CD11B, and CD62L. However, LPS-LDN (in vitro LDN) showed lower expression of CD63, CD184, and PD-L1 compared with LDN from patients (sepsis-LDN), suggesting a partial LPS impact on LDN generation. From the microscopic-based method (Wright’s staining in PBMC), sepsis-LDN demonstrated a mixed population of mature and immature cells with a good correlation with the flow-based analysis (Bland–Altman analysis and AUC). In conclusion, LDN in sepsis, partly generated by LPS activation, was associated with secondary infection and T cell suppression, mainly through the expression of PD-L1, which might be an immune suppression biomarker, especially with a less expensive microscopic-based method.

## Introduction

Sepsis, the response to severe infection, is a life-threatening organ dysfunction due to an aberrant host response, which is one of the important causes of death in the intensive care units (ICU). In the immunological aspect, severe sepsis might be due to the overwhelming responses, referred to as “sepsis hyperinflammatory responses”, or the inadequate immune responses, as called “sepsis-induced immune suppression”^1^. While cytokine storm during sepsis-induced hyperinflammatory causes septic shock with early mortality, immune suppression-induced nosocomial infection might occur at the later phase of sepsis^1,2^. Adjunctive immune therapies in sepsis depend on the direction of immune responses; for example, immunosuppressive drugs might be suitable for the hyper-inflammatory phase^3^, while immune-stimulatory molecules might be beneficial in sepsis immunosuppression^4^.

Then, the stratification of patients with sepsis in different immune statuses for using different targeted therapies might be a future personalized adjuvant immunotherapy for sepsis. Sepsis-induced immunosuppression impairs several components, including phagocytes (migration, phagocytosis, and bactericidal activities), antigen-presenting cells (APCs), and lymphocytes (apoptosis and anergy), leading to an enhanced susceptibility to secondary infections and increased mortality^5–7^. The direct evidence of immune suppression after sepsis is the fungal infections^8^ and reactivation of several latent viruses (cytomegalovirus, herpes, and Epstein-Barr)^9^ after sepsis. Interestingly, sepsis-induced immune suppression might be detectable in asymptomatic individuals with the increased susceptibility to infection, and molecular biomarkers are necessary. Because the clinical sign of sepsis-induced immune suppression remains highly undetectable and ambiguous by the clinical manifestation, specific biomarkers are necessary. Although the biomarkers for sepsis immune suppression, including an abnormality in the antigen-presenting cells (deceased HLA-DR) and T cell exhaustion (programmed cell death protein-1 and T-cell immunoglobulin and mucin-domain containing-3), are mentioned, other immune cells also have roles in sepsis-induced immune suppression^1,7^.

Neutrophils represent the most abundant white blood cells (WBC) mainly used for detection and elimination of organisms with high heterogeneity, plasticity, and adaptive immunity collaboration. As such, low-density neutrophils (LDN), the neutrophils identified in the peripheral blood mononuclear cells (PBMC) portion after the density gradient separation (Ficoll-Hypaque centrifugation), are identified in cancers with immune suppressive properties that might be correlated with “tumor-associated neutrophils” (the neutrophils that support tumor growth through the immune inhibition), also called “granulocytic myeloid-derived suppressor cells (gMDSC)^10^. In contrast, LDN in autoimmune diseases demonstrates the hyper-inflammatory properties that might be associated with the overwhelming immune responses^10^. Although LDNs have been extensively studied in various diseases, data on LDN in sepsis is still limited and might be correlated with immune suppression and stimulation, as mentioned in cancers and autoimmune diseases, respectively. Therefore, a better understanding of the impact of immune responses between different forms of neutrophils may provide beneficial prospects for future development of predictive biomarkers, and immune modulatory therapies targeting neutrophils. In the current study, we aimed to determine the characteristics and impacts of LDN in patients with sepsis.

## Material and method

### Study population

The Ethics Committees of the King Memorial Chulalongkorn Hospital (KMCH) approved the study (IRB No. 610/64) to use blood samples from the healthy volunteers and patients with sepsis with written informed consents according to the STROBE guideline. Then, patients with sepsis admitted to the intensive care unit (ICU) of KMCH between December 2021 and October 2023 and the healthy volunteers were recruited. The inclusion criteria were age > 18 years, diagnosed by at least 2 physicians, and sequential organ failure assessment (SOFA) scores higher than 2, while the exclusion criteria were pregnancy, hematologic diseases, neutropenia, the use of granulocyte-colony stimulating factor and immunosuppressive drugs, and organ transplantation history. The initial sepsis severity using the APACHE II and SOFA scores was performed at the enrollment. The catheter-related infection and major acute infectious events in the respiratory system, urinary tract, and digestive tract of each patient were noted during follow-up prospectively.

### Isolation of peripheral blood mononuclear cells (PBMC) and neutrophils

Heparinized blood samples were collected within 24 h after fulfilling the sepsis criteria (Sepsis-3) and processed within 2 h after the collection without refrigeration. The normal- and low-density neutrophils, NDNs and LDNs, respectively, were isolated using double-layered-density gradient centrifugation containing the Lymphoprep® (Stem Cell Technologies, Vancouver, Canada) density separation (upper part) and PolymorphPrep® (Serumwerk, Bernburg, Germany) density separation (lower part) at a ratio of 1:1:1. Both fractions were centrifuged at 800 g for 30 min at room temperature with brakeless deceleration. The PBMCs at the interface of the plasma and Lymphoprep® layers were carefully isolated, and the neutrophils were recovered from the intermediary layer (the interface between Lymphoprep® and PolymorphPrep®). The isolated PBMC and neutrophil fractions were washed in 1X deionized phosphate buffer solution (D-PBS) and eradicated contaminating red blood cells (RBCs) by 1X RBC lysis buffer (Biolegend, San Diego, CA, USA) before resuspension in the complete RPMI-based media, RPMI1640 culture media (Gibco, Waltham, MA, USA) containing 10% heat-inactivated fetal bovine serum (FBS) (Gibco, Waltham, MA, USA). The cell viability was observed by Trypan blue dye staining (Thermo Fisher Scientific, Waltham, MA, USA).

### Low-density neutrophil induction in vitro

The isolated neutrophils from gradient separation, as mentioned above, were incubated with the complete RPMI media. For low-density neutrophils (LDNs) in vitro induction, the isolated neutrophils (1 × 10^6^cells) were incubated with lipopolysaccharide (LPS) from *Escherichia coli* 026:B6 (Sigma-Aldrich, St. Louis, MI, USA) at various concentrations following a previous report^11^. After activation, stimulated neutrophils were re-centrifuged using the density gradient separation method before the retrieval of NDNs and LDNs from the upper and lower parts of the Lymphoprep® and PolymorphPrep®, respectively. The percentage of the induced LDNs was counted under a microscope and the cell characteristics were analyzed by flow cytometry (mentioned later).

### Flow cytometry

All analyses were measured using a FACS Canto II cytometer (BD Biosciences, Franklin Lakes, NJ, USA) with the FlowJo V10 (Ashland, DE, USA). Neutrophils and PBMC (5 × 10^5^ cells) were suspended in staining buffer (2% fetal bovine serum with 0.1% sodium azide in 1XD-PBS) and labelled with antibody panels for flow cytometric analysis. Antibodies used in the experiment were fluorescein isothiocyanate (FITC)-conjugated anti-CD66b (BD Biosciences, Franklin Lakes, NJ, USA), phycoerythrin (PE)-conjugated anti-CD63 (BD Biosciences, Franklin Lakes, NJ, USA), PE-conjugated anti CD279 (BD Biosciences, Franklin Lakes, NJ, USA), peridinin-Chlorophyll-Protein (Per-CP)-conjugated anti-CD14 (Biolegend, San Diego, CA, USA), PE-Cyanine 5 (cy5)-conjugated anti-CD184 (BD Biosciences, Franklin Lakes, NJ, USA), PE-Cyanine 7 (cy7)-conjugated anti-CD274 (BD Biosciences, Franklin Lakes, NJ, USA), PE-cy7-conjugated anti-CD182 (Biolegend, San Diego, CA, USA), allophycocyanin (APC)-conjugated anti-CD62L (BD Biosciences, Franklin Lakes, NJ, USA), APC-conjugated anti-CD45 (BD Biosciences, Franklin Lakes, NJ, USA), and APC-cy7-conjugated anti-CD11b (BD Biosciences, Franklin Lakes, NJ, USA). The cells were labeled for 30 min at 4 °C in the dark with antibodies before being fixed with 4.2% paraformaldehyde (BD Biosciences, Franklin Lakes, NJ, USA). Analysis was gated by the dot-plot analysis, and at least 25,000 cells were acquired per sample.

Apoptosis was quantified by annexin V-FITC and propidium iodide (PI) staining (BD Biosciences, Franklin Lakes, NJ, USA), with early apoptotic cells (annexin V + /PI-) and late apoptosis (annexin V + /PI +) using an apoptosis assay following the manufacturer’s instructions. After incubating for 20 min at 4 °C in the dark, cells were washed and measured. The reactive oxygen species (ROS) production was assessed by detecting fluorescence changes in the cells loaded with dihydroethidium (DHE) (Thermo Fisher Scientific, Waltham, MA, USA), following previous protocols^12^. Briefly, the cells (2.5 × 10^5^ cells) were resuspended in the complete RPMI-based media containing 2.5 μM DHE and incubated for 15 min at 37 °C in the dark. Cells were washed with cold D-PBS and resuspended in the cold 4.2% paraformaldehyde (BD Biosciences, Franklin Lakes, NJ, USA). Cells were stored cold in the dark until analyzed by flow cytometry using 485 nm (excitation; blue laser) and 520 nm (emission; PE) filters, gated by dot-plot analysis (10,000 cells were acquired per sample), and reported as mean fluorescent intensity (MFI). To analyze the phagocytic capacity, all cells were mixed with pHrodo S. aureus Bioparticle™ (Thermo Fisher Scientific, Waltham, MA, USA) before labeling with anti-CD66b antibody (a neutrophil biomarker) and being fixed with 4.2% paraformaldehyde (BD Biosciences, Franklin Lakes, NJ, USA). Phagocytosis was demonstrated as a percentage of the CD66b-positive cells with positive pHrodo S. aureus Bioparticle™.

### Human T cell isolation and proliferation assay

Isolated T cells in the suspension were captured by immunomagnetic negative selection using the EasySep™ Human T Cell Isolation Kit (Stem Cell Technologies, Vancouver, Canada) and the isolated CD3 + T cells were labeled with trypan blue (Thermo Fisher Scientific, Waltham, MA, USA) to detect cell viability. The T cell proliferation of PBMC or isolated CD3 + T cells was assessed by carboxyfluorescein succinimidyl ester (CFSE) (Thermo Fisher Scientific, Waltham, MA, USA) dilution as previously described^13^. Briefly, the proliferation index was calculated from the total number of divisions divided by the number of cells that underwent cell proliferation processes (http://docs.flowjo.com/vx/experiment-based-platforms/proliferation/plat-prolif-protocols/). After CFSE labeling, the isolated CD3 + T cells (1.0 × 10^5^ cells) or T cells in PBMC (containing 1.0 × 10^5^ CD3 + T cells) were seeded into a 96-well plate and stimulated with CD3/CD28 dyna-bead (Thermo Fisher Scientific, Waltham, MA, USA) using the unstimulated T cells (no dyna-bead) as the control according to the manufacturer’s protocol. After activation for 4 days, the cells were harvested and were stained with APC-conjugated anti-CD3 antibody (BD Biosciences, Franklin Lakes, NJ, USA) and analyzed by flow cytometry as previously described^13^.

### Wright–Giemsa stain assay

The PBMC and neutrophils portion after gradient separation were smeared, naturally dried, stained with the Wright-Giemsa solution (Merck, Darmstadt, Germany) at room temperature for 1–2 min, mixed with phosphate buffer solution (pH 7.2), and washed with sterile water. The results of cell staining were observed using a bright field microscope (Nikon, Shinagawa, Tokyo, Japan).

### Statistical analysis

GraphPad Prism 8.0 (GraphPad Software, Inc.) was used for statistical analysis and graph presentation using the student’s t-test or Mann–Whitney U test and one-way analysis of variance (ANOVA) with Tukey’s analysis or Kruskal–Wallis for the 2- and 3-group comparisons, respectively. A *p*-value < 0.05 was considered a statistically significant difference. Kaplan–Meier analysis and receiver operating characteristic (ROC) curve were used to predict the efficacy of the circulating LDN for determination of secondary infection at 45 day after the enrollment. The agreement between the accumulation of LDN in PBMC measured by flow cytometry and microscopy was assessed by Bland Altman analysis^14^.

## Result

### Demographic data of the study population

Patients in the intensive care unit (ICU) with sepsis (n = 34) and without sepsis (n = 18) were enrolled. From these, ten patients with sepsis were excluded due to a history of drug administration and organ transplantation, while eight patients without sepsis were excluded due to unclear diagnosis. Then, the study included 24 eligible ICU patients with sepsis and 10 patients without sepsis, along with 20 healthy volunteers. The clinical and biological characteristics of all groups are detailed in (Table 1).

**Table 1:** Demographic data.

	Sepsis (n = 24)	ICU patients (n = 10)	Healthy (n = 20)
Age, mean (SD)	53.9 (16.2)	45.5 (21.5)	39.6 (14.5)
Gender, male (%)	10 (41.7)	5 (50)	10 (50)
ICU days, median (IQR)	10 (6–14)	14.5 (8.5–19)	N/A
Hospital days, median (IQR)	17.5 (10.5–34)	23 (11–53)	N/A
SOFA score	8 (4–9)	5 (4–8)	N/A
APACHE II score	16 (14–22)	16 (8–20)	N/A
Laboratory			
Hemoglobin (g/dL), mean (SD)	8.50 (1.01)	8.73 (1.63)	12.15 (1.20)
WBC count (× 10^9^/L), mean (SD)	11.26 (6.19)	9.52 (5.72)	5.36 (1.44)
Absolute neutrophil count (× 10^9^/L), mean (SD)	8.53 (4.77)	7.01 (4.47)	2.92 (1.02)
Absolute lymphocyte count (× 10^9^/L), mean (SD)	1.46 (0.92)	1.91 (0.77)	1.72 (0.58)
Hemoculture positive, n (%)	14 (58.3%)	N/A	N/A
Site of infection			
Respiratory	15 (62.5)	4 (40)	N/A
Abdominal	1 (4.2)	N/A	N/A
Others	8 (33.3)	N/A	N/A

*SD* standard deviation, *IQR* internal quartile range, *ICU* intensive care unit, *SOFA* the sequential organ failure assessment, *APACHE* the acute physiology and chronic health evaluation, *WBC* white blood cells, *N/A* not applicable.

### LDN expansion in patients with sepsis

**Fig. 1:**
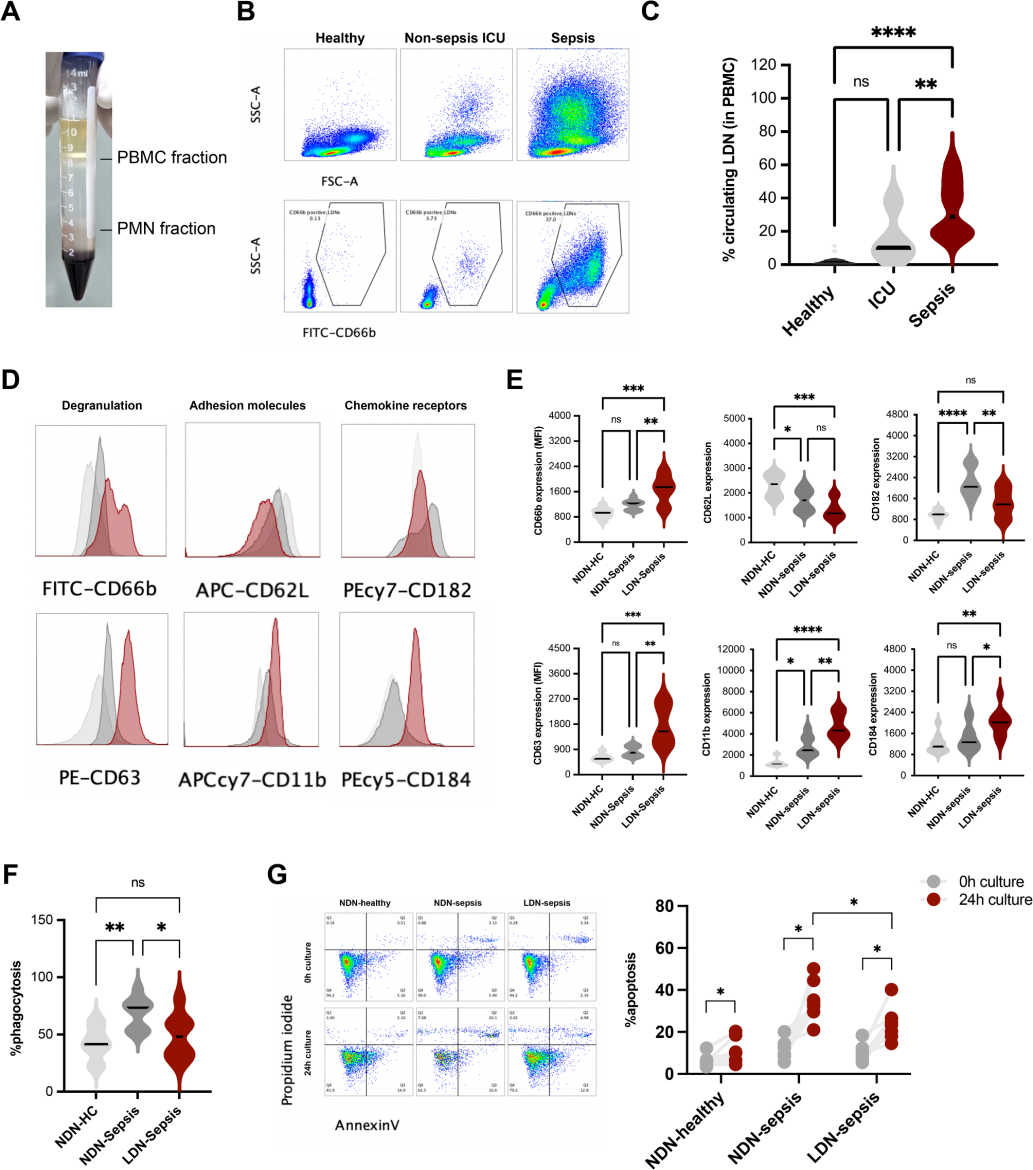
The representative pictures of the gradient separation with the peripheral blood mononuclear (PBMC) fraction and polymorphonuclear cells (PMN; neutrophil) fraction (**A**) and characteristics of the samples, including healthy control, sepsis, and non-sepsis in the intensive care unit (ICU), as indicated by the percentage of LDN (the neutrophils in the PBMC fraction) through the representative flow cytometry dot-plot patterns with graphs (**B**,**C**), are demonstrated. Characteristics of normal-density neutrophils from healthy control (NDN-HC) and from sepsis (NDN-sepsis) and LDN from sepsis (LDN-sepsis), as indicated by the surface markers through the representative flow cytometry histogram with graphs of CD66b (specific granule), CD62L (L-selectin), CD182 (chemotaxis-associated CXCR2), CD63 (primary granule), CD11b (adhesion molecule), and CD184 (Fusin or CXCR4) (**D**,**E**), percentage of phagocytosis (**F**), and apoptosis (representative flow cytometry dot plot and graphs using annexin V and propidium iodide) (**G**) are also demonstrated. *ns* non-significance; *p < 0.05; **p < 0.01; ***p < 0.001; ****p < 0.0001.

The density gradient centrifugation of peripheral blood was performed to separate PBMC and neutrophil fractions using (Fig. 1A), and the flow cytometry gating from the PBMC fraction using CD66b (a marker for neutrophils) (Fig. 1B) demonstrated more prominent LDN (CD66b-positive cells in the PBMC) in the patients with sepsis compared with the non-sepsis (ICU) and healthy control (Fig. 1C). In comparison with the normal density neutrophils (NDN) from the same patients (NDN-sepsis) or healthy control (NDN-HC), LDN from sepsis (LDN-sepsis) exhibited higher expression of the degranulation markers, including CD66b (specific granule marker) and CD63 (primary granule marker), CD11b (integrin alpha M), and CD184 (Fusin or CXCR4), with lower expression of CD62L (L-selectin) and CD182 (chemotaxis-associated CXCR2), which obviously differed from other groups (Fig. 1D,E). Meanwhile, an impairment in phagocytic activity was demonstrated in LDN-sepsis when compared with the NDN-sepsis or NDN-HC (Fig. 1F). Notably, the abundance of LDN from healthy control was too low to separate for further evaluation. Although all groups of neutrophils (LDN-sepsis, NDN-sepsis, and NDN-HC) underwent apoptosis, there was less apoptosis in LDN-sepsis compared with NDN-sepsis (Fig. 1G).

### Sepsis-related LDN, an indicator for the secondary infection in sepsis

**Fig. 2:**
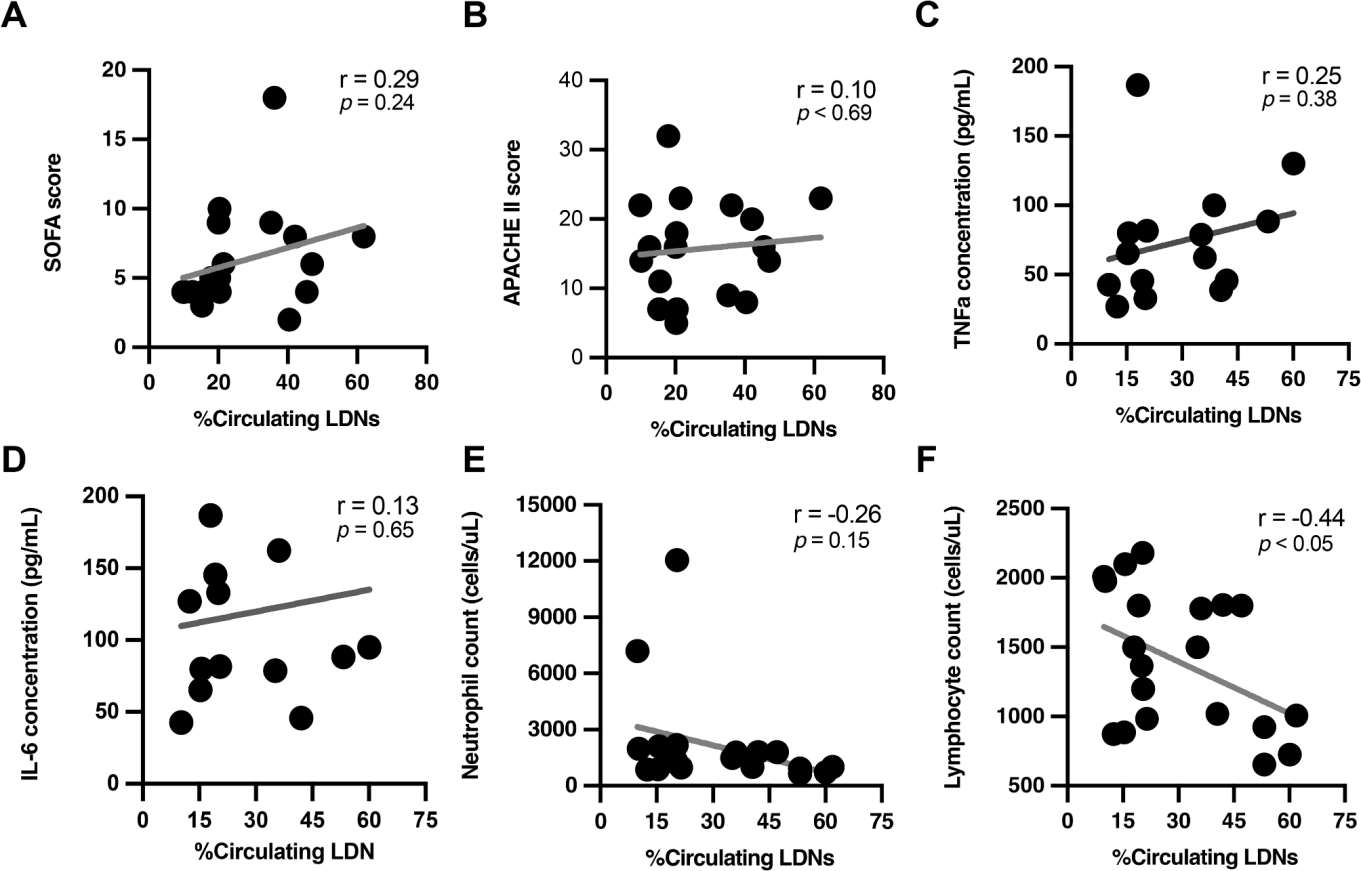
The correlation between the percentage of low-density neutrophils (LDN; neutrophils in the PBMC fraction) from patients with sepsis and several indicators, including the Sequential Organ Failure Assessment (SOFA) score (**A**), the acute physiology and chronic health evaluation (APACHE) II score (**B**), serum TNFa (**C**), serum IL-6 (**D**), blood neutrophil counts (**E**), and blood lymphocyte count (**F**), is demonstrated.

**Fig. 3:**
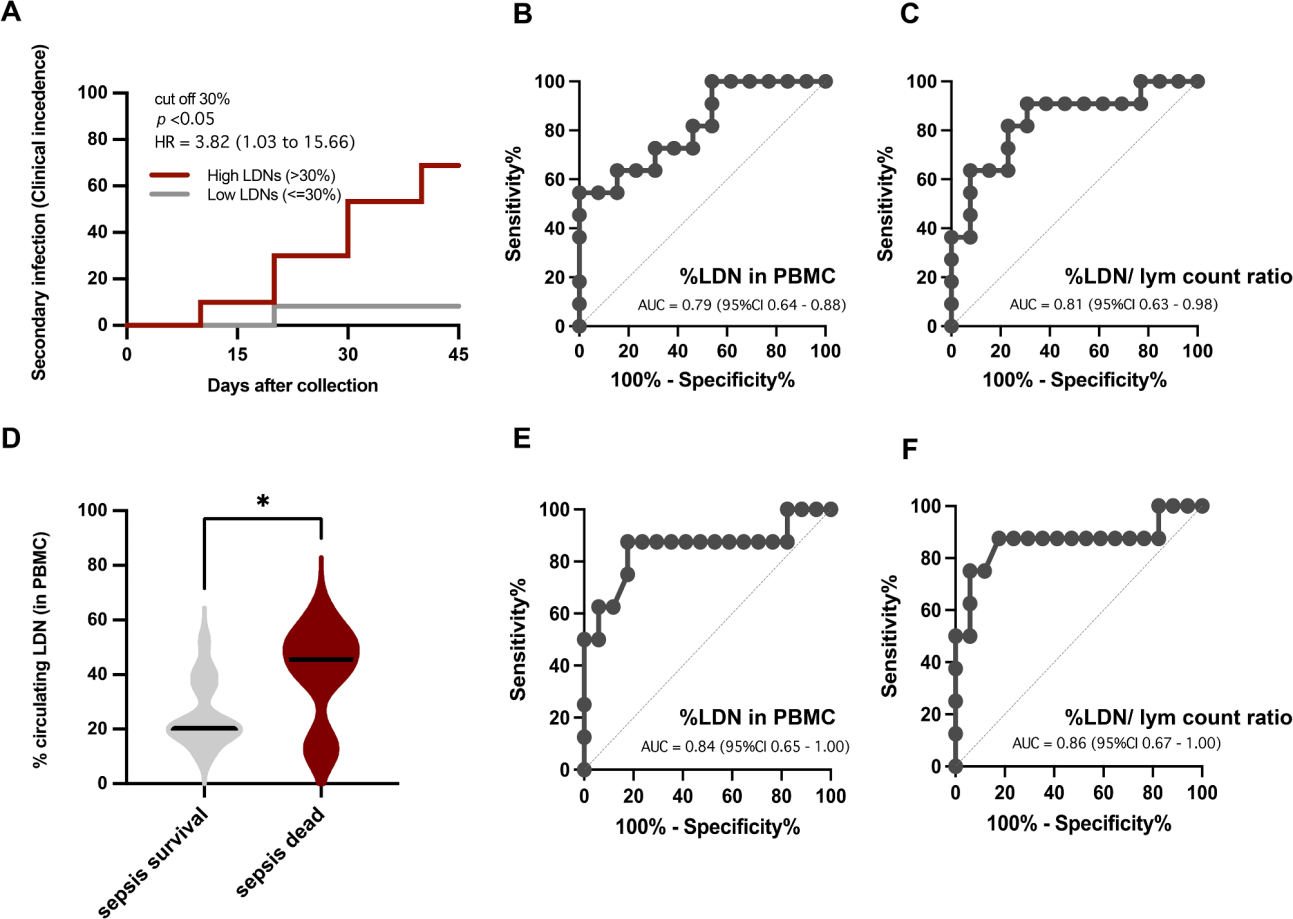
Characteristics of patients with patients with sepsis with LDNs higher or lower than 30% (red or gray lines, respectively) as indicated by the percentage of secondary infection at 45 days (**A**), the receiver operating characteristics-area under the curve (ROC-AUC) for the prediction of secondary infection using LDN (% LDN in the PBMC fraction) alone (**B**), and LDN divided by lymphocyte count (% LDN in the PBMC fraction/lymphocyte count) (**C**). The abundance of LDN in patients with recorded death and survival at 45 days of sepsis (**D**) and the ROC-AUC for the prediction of mortality using LDN alone (**E**) and LDN divided by lymphocyte count (**F**) are also demonstrated. *p < 0.05.

The correlation between the level of LDN and several sepsis parameters was performed to evaluate the roles of LDN in sepsis. As such, there was a trend of positive correlation (not significant difference) between the LDN and clinical outcomes of patients with SOFA and APACHE II scores, TNF-a, and IL-6 (Fig. 2A–D). Meanwhile, a negative correlation between LDN and lymphocyte count with a trend of negative correlation to the neutrophil count was demonstrated (Fig. 2E,F). Using the median value of patients with sepsis as a cut-off value, septic patients with high LDN (LDN > 30% in the PBMC) (n = 8; %LDN, median (IQR) = 43.7 (36.2–53.2)) demonstrated a higher incidence of secondary infection (an important indicator of sepsis-induced immune suppression) compared to patients with low levels of LDN (n = 9; %LDN, median (IQR) = 16.6 (10.8–20.9)), as the hazard ratio (HR) of 3.82 with a 95% confidence interval (95% CI) of 1.03 to 15.66 (Fig. 3A). The Receiver Operating Characteristics-Area Under the Curve (ROC-AUC) demonstrated the efficacy of LDN level to predict the incidence of secondary infection within 45 days with the area under the curve (AUC) of 0.79 (95% CI 0.64–0.88) (Fig. 3B), which was improved into the AUC of 0.81 (95% CI 0.63–0.98) with the use of % LDN/lymphocyte count ratio (Fig. 3C). For mortality rate prediction, septic patients with ICU death (n = 7, %LDN, median (IQR) = 45.5 (40.3–53.2)) exhibited a higher level of LDN compared with the survivors (n = 17; %LDN, median (IQR) = 20.5 (19.1–29.3)) (Fig. 3D). The AUC to predict ICU mortality was 0.84 (95% CI 0.65–1.00) and 0.81 (95% CI 0.63–0.98) using LDN and % LDN/lymphocyte count ratio, respectively (Fig. 3E,F). Due to the correlation between low lymphocyte count and an increased infection susceptibility in sepsis^15^, LDN may play an essential role in sepsis-induced immune suppression.

### Suppression of T cell function in sepsis by LDN

**Fig. 4:**
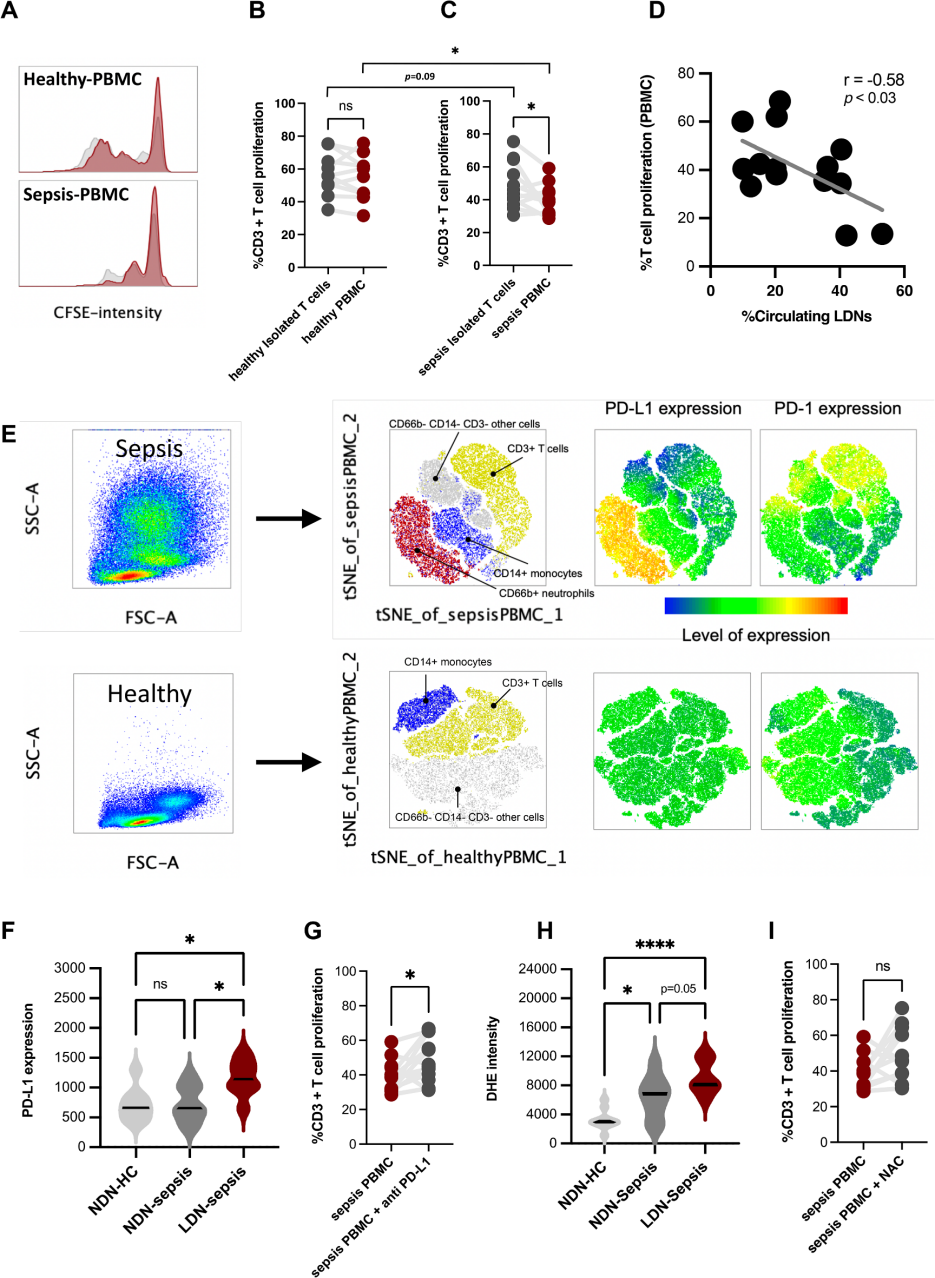
Characteristics of peripheral blood mononuclear cells (PBMC; red color) and isolated CD3 positive T cells (grey color) from healthy control and patients with sepsis are indicated by the histogram of carboxyfluorescein succinimidyl ester (CFSE; a fluorescent staining for T cell proliferation) (**A**), and the graphs from healthy control (**B**) and from patients with sepsis (**C**) and correlation of percentage of LDN with T cell proliferation (**D**). The representative picture of flow cytometry gating and t-distributed stochastic neighbor embedding (tSNE) visualization of PBMC staining from patients with sepsis and healthy controls demonstrated the separation of neutrophils (CD66b positive; LDN), monocytes (CD14 positive), T cells (CD3 positive), and other cells with expression of program cell death ligand-1 (PD-L1) and program cell death-1 (PD-1) (**E**) is also demonstrated. Characteristics of normal density neutrophils of healthy control (NDN-HC) or sepsis (NDN-sepsis) and low-density neutrophils from sepsis (LDN-sepsis), as indicated by PD-L1 expression (**F**) with the percentage of T cell (CD3 positive cells) proliferation in PBMC from sepsis (sepsis PBMC) with or without anti-PD-L1 (**G**), are shown. Reactive oxygen species (ROS) as evaluated by dihydroethidium (DHE) staining in NDN-HC, NDN-sepsis, and LDN-sepsis (**H**) with the percentage of T cell proliferation in sepsis PBMC with and without a ROS scavenger using N-acetyl cysteine (NAC) are also shown. *p < 0.05.

Because of the well-known impacts of T cell suppression in sepsis-induced immune suppression^16^, the in vitro experiments were performed. As such, T cell stimulator (anti-CD3 and anti-CD28 dyna-bead) was incubated in the PBMC and isolated CD3 + T cells from patients with sepsis and healthy control for 4 days, and T cell proliferation was evaluated using carboxyfluorescein succinimidyl ester (CFSE) staining (Fig. 4A). In sepsis, the stimulated T cell in PBMC (containing T cells and LDN), but not isolated T cells (T cells alone without LDN), exhibited a decrease in T cell proliferation, while T cell proliferation in healthy control was similar between the isolated T cells and PBMC (Fig. 4B,C). Also, T cell proliferation in sepsis PBMC (high LDN in sepsis PBMC) was lower than the healthy control PBMC (very little LDN in PBMC), while T cell proliferation in the isolated T cells (no LDN) of sepsis and control groups was similar (Fig. 4B,C). Notably, the negative correlation between % circulating LDN and T cell proliferation rate in PBMC was also found (Fig. 4D). These results implied the capacities of LDN to suppress T cell activities.

In cancer research, LDN are characterized by their suppressive capability on T cell response via expression of PD-L1 and ROS generation to interrupt T cell receptor (TCR) complex signaling^17,18^. With the t-Distributed Stochastic Neighbor Embedding (tSNE) visualization of PBMC staining in sepsis, the figures demonstrated that LDN (CD66b-positive cells in the PBMC fraction) are a strong expression of surface PD-L1 population, while PD1-positive cells were CD3 + T cells (Fig. 4E). Moreover, LDN-sepsis prominently exhibited PD-L1 expression compared with the normal-density neutrophils (NDN) of sepsis and healthy control (Fig. 4F). Additionally, the role of PD-L1 in LDN-induced T cell suppression was supported by the neutralization of T cell suppression with anti-PD-L1 antibody (Avelumab; an anti-PD-L1 neutralizing antibody), which increased T cell proliferation in sepsis-PBMC (Fig. 4G). Likewise, production of reactive oxygen species (ROS), as indicated by dihydroethidium (DHE), in LDN-sepsis was increased when compared with NDN from both sepsis and healthy control (Fig. 4H). Also, both NDN and LDN from sepsis were more prominent than NDN in healthy control (Fig. 4H). However, a ROS scavenger (NAC; N-acetyl cysteine) showed only a trend to neutralization of T cell suppression in PBMC of sepsis (Fig. 4I). This study suggested that PD-L1:PD-1 axis may be the crucial mechanism of LDN-induced T cell suppression in sepsis.

Interestingly, the incubation of lipopolysaccharide (LPS) with the isolated neutrophils from healthy volunteers induced LDNs (LPS-LDNs) in dose and time dependent manner with different surface immunophenotypes from healthy control neutrophils (NDN-HC), normal-density neutrophil-derived from LPS (LPS-NDN-HC), and LDN from patients with sepsis (LDN-sepsis) (Supplement Fig. 1A–H). Sepsis-induced LDNs can partly be generated by the induction of endotoxemia; however, some characteristics of LDN-sepsis (CD63, CD184, and PD-L1) might be induced by other factors.

### Measurement of LDN using a conventional bright field microscope

**Fig. 5:**
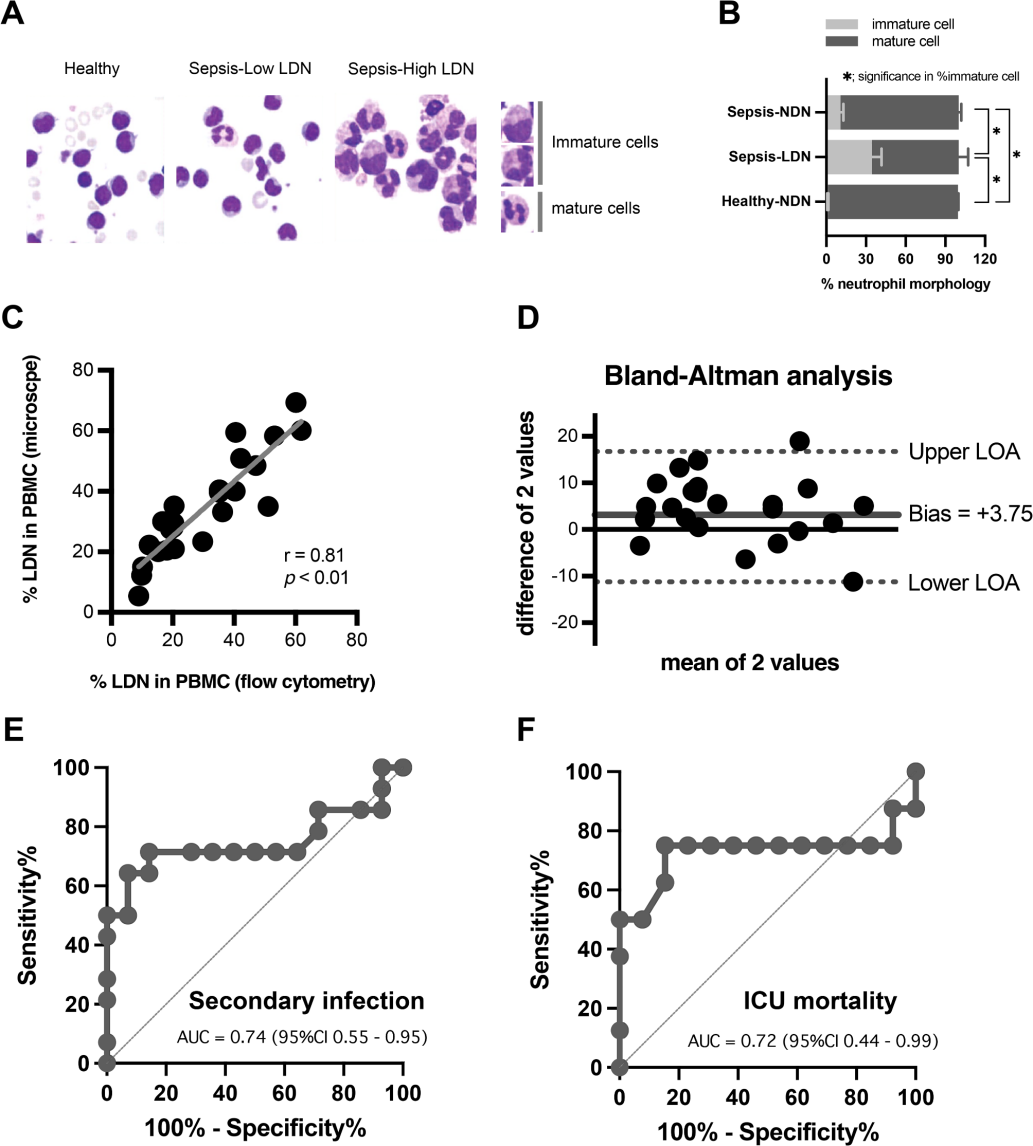
Representative pictures of peripheral blood mononuclear cells (PBMC fraction) from healthy volunteers (no neutrophils) and from sepsis with low or high levels of low-density neutrophils (sepsis-Low LDN and sepsis-High LDN) (**A**) and the percentage of neutrophils maturity as determined by the morphology in Wright Giemsa stain of neutrophils from sepsis (normal- and low-density; sepsis-NDN and sepsis-LDN) and from healthy volunteers (normal- and low-density; Healthy-NDN and Healthy-LDN) (**B**) are demonstrated (n = 8–12/group). The correlation of LDN in PBMC fraction as determined by the percentage of neutrophils using Wright’s stain (microscope-based method) versus using flow cytometry analysis (**C**), Bland–Altman analysis (**D**), and the receiver operating characteristics-area under the curve (ROC-AUC) for the prediction of secondary infection (**E**) and mortality (**F**) using LDN from the microscope-based method are also demonstrated.

Due to the more sophisticated equipment of flow cytometry over the microscope-based method^19^, the potential to measure LDN accumulation by conventional bright field microscope (gradient separation and determining percentage of LDN in PBMC with Wright Giemsa stain) for applying in the unit where flow cytometer was unavailable. Maturation of neutrophils from microscopic analysis using nucleus morphology. While the regular NDN appeared to be a homogenous population of mature, multi-segmented neutrophils (multilobed nuclei), LDNs were heterogeneous and contained mature-segmented neutrophils and immature neutrophils (myelocyte-like or band-shaped nuclei) (Figs. 5A,B). Accordingly, there was an acceptable correlation between LDN from microscope- and flow cytometry-based methods (Fig. 5C), and the difference between these methods with the Bland–Altman analysis indicated the mean difference at 3.75% with the limit of agreement at 12.5–16.75% with 95% of the differences being within ± 1.96 SD (Fig. 5D). Finally, the ROC-AUC curve also showed the efficacy of LDN level measured by microscopy with Wright Giemsa staining as the AUC at 0.74 (95% CI 0.55–0.95) and 0.72 (95% CI 0.44–0.99) to predict the incidence of secondary infection and mortality, respectively (Fig. 5E,F). Hence, the LDN measurement using the microscope-based method might be suitable for hospitals with limited resources.

## Discussion

In patients with sepsis who survived septic shock and sepsis hyper-inflammatory responses, nosocomial infection from sepsis-induced immune suppression is an important complication with high morbidity and mortality, especially in patients with persistent lymphopenia and T-cell exhaustion^20,21^. Although neutrophils are very important for microbial control, as indicated by neutropenic severe infection, hyper-activated neutrophils are partly responsible for tissue damage in sepsis^22^. Interestingly, immunosuppressive impacts of the mature neutrophils from humans are currently demonstrated as follows: i) a suppression of T-cell proliferation in an integrin Mac-1- and ROS-dependent manner^23^, ii) an interference of T helper 1 responses from serine proteases and neutrophil elastase through the cleavage of monocyte CD14 and several co-stimulatory molecules of dendritic cells^24^, and iii) the tumor-associated neutrophils in cancers^25^. Possibly, the immune suppression properties of neutrophils might partly be one of the control systems to reduce the initial overwhelming bactericidal activity of neutrophils, allowing the development of adaptive immune responses to control the organisms. In regular inflammatory responses, neutrophil accumulation is observed in the first week before the influx of other immune cells, especially macrophages^26^. In sepsis, a detailed assessment of the immune system is needed to observe the change of neutrophil activities along with sepsis progression due to the phenotypic heterogeneity and functional diversity.

Low-density neutrophils (LDN) are previously reported as a “contaminant” in PBMC fraction, which currently are demonstrated as a unique neutrophil population in several conditions, including cancers, chronic inflammation, chronic hepatitis, HIV infection, and some physiological conditions (pregnancy and infancy)^27,28^. Here, very little LDN was detected in the healthy control, but it was expanded along with sepsis severity, possibly due to the alteration of the sepsis microenvironment (such as LPS stimulation). Then, LDN might be beneficial as a biomarker for sepsis severity. The negative correlation between LDN and lymphocyte parameters, including cell count and cell proliferation (the important mechanisms of sepsis-induced immune suppression), also supported the possible LDN-induced immune suppression leading to secondary infection and the increased mortality. The AUC of LDN to predict sepsis-induced secondary infections was greater than 0.7 (an adequate level to use in the real clinical situation)^29^, and the use of LDN together with lymphocyte count improved the AUC for prediction of nosocomial infection and mortality. Therefore, LDNs could serve as a practical biomarker for immune suppression with the less expensive microscopic-based method. This method is simpler than other biomarkers, such as monocyte HLA-DR (mHLA-DR), which requires multi-color flow cytometric analysis for the measurement^30^.

In comparison with normal-density neutrophils (NDN), LDN more prominently expressed CD66b (specific granules) and CD63 (primary granules), suggesting a possible dominant degranulation property, while higher CD184 (CXCR4) with lower CD182 (CXCR2), imply the neutrophil trafficking regulation (bone marrow release) through the CXCR2/CXCR4 antagonistic property^31^. More importantly, the higher expression of PD-L1 in LDN over NDN (high PD-L1 in CD66b-positive cells in PBMC; Fig. 4E) might be responsible for the lower apoptosis of LDN than NDN (anti-apoptotic effect of PD-L1) (Fig. 1G)^32^ and the T cell suppression property, similar to the LDN in cancers^17,33,34^. Interestingly, the T cell suppression property of LDN was attenuated by anti-PD-L1 antibody (Avelumab) treatment, supporting the benefits of anti-PD-L1 neutralizing antibody in patients with sepsis^35–37^. Thus, NDN in sepsis might be a control of proinflammatory status, but the LDN counterbalance is too strong, which finally directs immune responses into the immune suppression. Although we suggested PD-L1: PD-1 axis as an underlying mechanism of LDN-mediated T cell suppression, other mechanisms, such as ROS production, could also be involved in sepsis-immune suppression. However, ROS levels in sepsis-LDN and sepsis-NDN were similar, and the antioxidant (NAC) could not attenuate LDN-induced T cell suppression, perhaps due to the limited antioxidant effect of NAC against sepsis. Given together, this study suggested that the PD-L1: PD-1 axis and ROS generation may be the crucial mechanisms of LDN-induced T cell suppression in sepsis, similar to LDN in the cancer-induced immune suppression referred to as “granulocytic myeloid-derived suppressor cells (gMDSCs). Moreover, elevation of degranulation measured by an increase in CD66b and CD63 on the cell surface was also interesting. Perhaps LDN can release some suppressive molecules, such as arginase I and transforming growth factor beta (TGF-b), to the cell environment. Further studies in this topic are interesting.

Although LDN have been described as a unique population of neutrophils that presented in the PBMC fraction after density centrifugation, the source of LDN in patients with sepsis remained unclear with several hypotheses. Several theories may shed light on the source. First, sepsis LDN might be immature neutrophils (lower density due to the immature granules and nuclei) due to the increased demand for microbial control, which is consistent with the high plasma G-CSF to enhance myelopoiesis^38^. The increased immature neutrophils in sepsis might be due to the early disruption of myeloid maturation or the deviation of neutrophil differentiation due to the persistent inflammatory environment, as previously mentioned in infection, cancer, and systemic inflammatory response syndrome (SIRS)^38,39^. Due to the limited abundance of LDNs in the health control, the comparison between control LDNs and sepsis LDNs is difficult. Further studies with the comparison between LDNs from non-infection causes and from sepsis will be interesting. Second, LDN might be the regular neutrophils (NDN) that are activated by some factors in blood circulation^40^, partly to enhance the degranulation property, which is supported here through the higher CD66b and CD63 (degranulation markers) of LDN over NDN (Fig. 1E) and the induction of LDN by LPS (Supplement Fig. 1A,B). This theory is also supported by the alteration of NDN into immunosuppressive LDN by tumor growth factor-beta in an animal model^41^. However, LDN from LPS activation (LPS-LDN) was different from the natural LDN in patients with sepsis. There was lower expression of PD-L1, CD63, and CD184 in LPS-LDN compared with the natural LDN (Supplement Fig. 1D,G,H), possibly because of other non-LPS proinflammatory molecules in sepsis^42,43^. Other additional factors to LPS might be required to make the characteristics of LPS-LDN closer to sepsis-LDN. Third, some experimental factors (continuous centrifugation and washing) might alter NDN into LDN partly through activating cell degranulation^44^. Hence, we immediately separated PBMC and NDN after blood collection without refrigeration to eliminate this factor in our study. Then, we hypothesize that sepsis-LDN consists of both immature cells and the NDN alteration from some activators, including LPS, and the sepsis-LDN exhibits T cell suppression property through the expression of PD-L1, partly resulting in immune suppression and enhanced infection susceptibility. For the clinical application, we proposed using gradient separation and a simple Wright’s stain to determine the percentage of LDN in the PBMC fraction, which will be inexpensive enough to use LDN as another sepsis parameter even in the situation with limited resource.

Finally, several limitations should be mentioned. First, the conclusion was based on a single-center analysis with a limited number of patients. The multicenter, larger cohorts with patients in different stages of sepsis and treatments on sepsis-LDN are required. Second, more sophisticated and detailed mechanistic experiments are required for the solid conclusions on the origin and development of LDN. Third, the separation of LDN was based on the gradient separation; however, the development of whole blood-based protocols for the identification of biomarkers might be beneficial for future applications (diagnostic and prognostic biomarkers). Fourth, the non-different ROS between LDN-sepsis and NDN-sepsis is determined only by DHE (Fig. 4H), which is oxidized by superoxide into 2-hydroxyethidium (2-OH-E +) with red fluorescent color^45^. The use of a probe that targets ROS in general, such as the oxidation of 2’,7'-dichlorofluorescein (DCFH) into 2’,7'-dichlorofluorescein (DCF)^46^ or the CellRoxTM probe^47^ might demonstrate some differences. Due to the positive DHE, the stimulated neutrophils activate NADPH oxidase (NOX) to generate superoxide, which acts as a precursor of hydrogen peroxide, hydrogen perchlorate (HOCL), and other ROS molecules^45,48^. Finally, this study did not directly compare the effectiveness between LDNs and other biomarkers, such as mHLA-DR. More studies are warranted.

In conclusion, the presence of neutrophils in the PBMC fraction, referred to as “low-density”, possibly consists of the immature cells and activated neutrophils with immune suppression properties, partly through increased expression of PD-L1 and T cell suppression, that could be used as a biomarker for the prediction of secondary infection and sepsis mortality. Further studies on the correlation of LDN abundance with T cell suppression and/or the responses to anti-PD-L1 are interesting.

## Supplementary Information

Supplementary Information.

## Data Availability

The datasets used and/or analyzed during the current study are available from the corresponding author on reasonable request.
