# Supplementary material for: Immune suppressive activities of low-density neutrophils in sepsis and potential use as a novel biomarker of sepsis-induced immune suppression
Source: Sci Rep. 2025 Mar 19;15:9458. doi: 10.1038/s41598-025-92417-7 (PMC11923122; doi:10.1038/s41598-025-92417-7)
Supplement: Supplementary file 1 — Supplementary Information. [file 41598_2025_92417_MOESM1_ESM.pptx]

## Slide 1
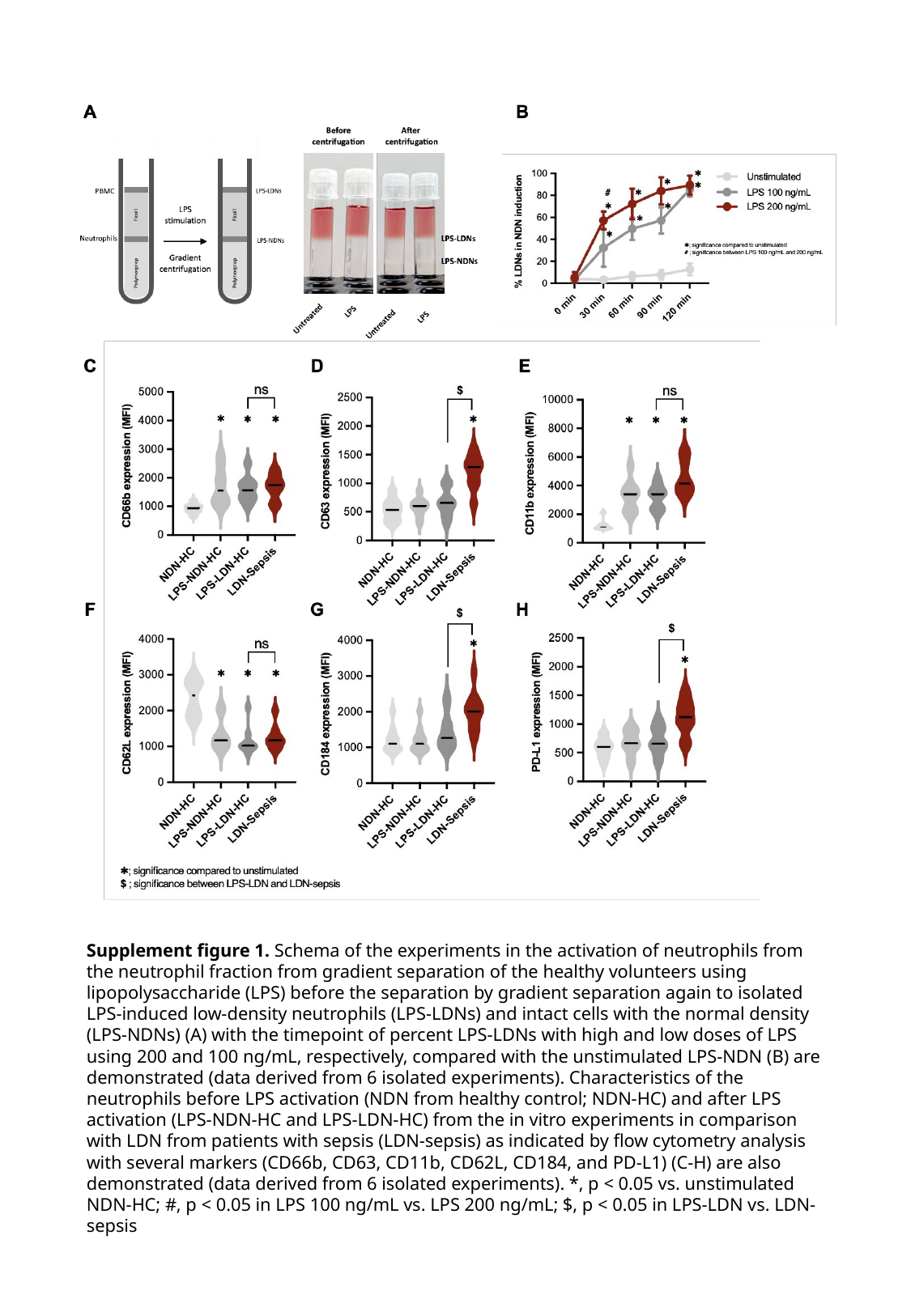

Supplement figure 1. Schema of the experiments in the activation of neutrophils from the neutrophil fraction from gradient separation of the healthy volunteers using lipopolysaccharide (LPS) before the separation by gradient separation again to isolated LPS-induced low-density neutrophils (LPS-LDNs) and intact cells with the normal density (LPS-NDNs) (A) with the timepoint of percent LPS-LDNs with high and low doses of LPS using 200 and 100 ng/mL, respectively, compared with the unstimulated LPS-NDN (B) are demonstrated (data derived from 6 isolated experiments). Characteristics of the neutrophils before LPS activation (NDN from healthy control; NDN-HC) and after LPS activation (LPS-NDN-HC and LPS-LDN-HC) from the in vitro experiments in comparison with LDN from patients with sepsis (LDN-sepsis) as indicated by flow cytometry analysis with several markers (CD66b, CD63, CD11b, CD62L, CD184, and PD-L1) (C-H) are also demonstrated (data derived from 6 isolated experiments). *, p < 0.05 vs. unstimulated NDN-HC; #, p < 0.05 in LPS 100 ng/mL vs. LPS 200 ng/mL; $, p < 0.05 in LPS-LDN vs. LDN-sepsis
